# Complete Genome Sequences of SARS-CoV-2 Strains Detected in Malaysia

**DOI:** 10.1128/MRA.00383-20

**Published:** 2020-05-14

**Authors:** Yoong Min Chong, I-Ching Sam, Sasheela Ponnampalavanar, Sharifah Faridah Syed Omar, Adeeba Kamarulzaman, Vijayan Munusamy, Chee Kuan Wong, Fadhil Hadi Jamaluddin, Han Ming Gan, Jennifer Chong, Cindy Shuan Ju Teh, Yoke Fun Chan

**Affiliations:** a Department of Medical Microbiology, Faculty of Medicine, University of Malaya, Kuala Lumpur, Malaysia; b Department of Medical Microbiology, University of Malaya Medical Centre, Kuala Lumpur, Malaysia; c Department of Medicine, Faculty of Medicine, University of Malaya, Kuala Lumpur, Malaysia; d Department of Anaesthesiology, Faculty of Medicine, University of Malaya, Kuala Lumpur, Malaysia; e GeneSeq Sdn Bhd, Rawang, Selangor, Malaysia; DOE Joint Genome Institute

## Abstract

We sequenced four severe acute respiratory syndrome coronavirus 2 (SARS-CoV-2) genomes from Malaysia during the second wave of infection and found unique mutations which suggest local evolution. Circulating Malaysian strains represent introductions from different countries, particularly during the first wave of infection. Genome sequencing is important for understanding local epidemiology.

## ANNOUNCEMENT

**FIG 1:**
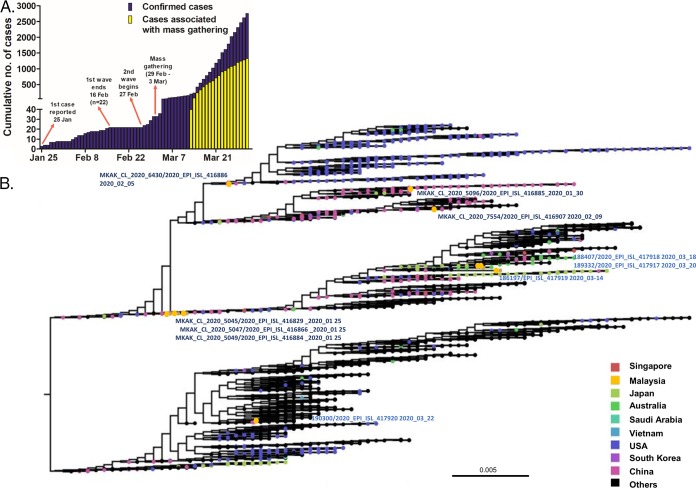
(A) Cumulative cases in Malaysia until 31 March 2020. There have been two waves of infection, separated by 11 days during which no cases were reported. Cases of the first wave were associated mainly with travel from China and Singapore. About 48% of the cases in the second wave were associated with a religious mass gathering. (B) Maximum likelihood phylogenetic tree built with FastTree and based on the complete genomes of 2,364 sequences available at GISAID on 29 March 2020. The 10 Malaysian genomes are named and comprise six genomes from the first wave (dark blue) and the four genomes sequenced in this study (second wave, blue). The date of sample collection is recorded at the end of each strain name. The tree was midpoint rooted.

Severe acute respiratory syndrome coronavirus 2 (SARS-CoV-2) belongs to the family *Coronaviridae* and the genus *Betacoronavirus* and has caused a pandemic of coronavirus disease (COVID-19). As of 31 March 2020, Malaysia had 2,766 confirmed cases with 43 deaths (1). To obtain a preliminary understanding of SARS-CoV-2 molecular epidemiology in Malaysia, we performed complete genome sequencing of SARS-CoV-2 strains collected directly from nasopharyngeal swabs from four patients (186197, 188407, 189332, and 190300) in Kuala Lumpur, Malaysia, between 14 and 22 March 2020 (Fig. 1A).

Viral RNA was extracted from the samples using a QIAamp viral RNA minikit (Qiagen, Germany) and amplified according to the ARTIC nCoV-2019 protocol (2). Briefly, cDNA was synthesized using a SuperScript IV first-strand synthesis system (Invitrogen, USA) with random hexamers. The ARTIC v1 primers were divided into two pools of 49 primer sets for PCR using Q5 high-fidelity DNA polymerase (NEB, USA). Overlapping amplicons of 400 bp were combined and purified using sample purification beads (SPB) (Illumina, USA), quantified with a Qubit 3.0 fluorometer, and used for library preparation. Nextera DNA Flex libraries were sequenced using iSeq 100 reagent (Illumina) on the iSeq 100 system (Illumina) with output of 2 × 100-bp paired-end reads and 4 million expected paired reads. Sequencing of one strain (186197) was also performed using a Nanopore MinION and ligation sequencing kit (SQK-LSK109) according to the Oxford Nanopore Technologies (ONT) standard protocol (ONT, UK). Briefly, purified amplicons were sequenced in an R9.4 flow cell and run for 30 min.

The iSeq raw FastQ files were analyzed using Geneious Prime 2020 (Biomatters, New Zealand). The average number of raw paired reads obtained from iSeq was 1.8 million (Table 1). Paired reads were trimmed for quality using default parameters and mapped to reference strain Wuhan-Hu-1 (GenBank accession number MN908947) with the Geneious mapper. About 94% of the reads were mapped, except for strain 186197 (25.2%). The average depth of coverage for iSeq was 5,000×, except for strain 186197, which had only 1,000× coverage (Table 1). Therefore, the consensus sequence for strain 186197 was mapped from a combination of iSeq (359,453 paired reads) and MinION (23,390 reads) sequencing with Geneious mapper using default parameters. The four genome sizes ranged from 29,486 to 29,898 bp with GC contents of 36.6 to 37.9% (Table 1). Multiple sequence alignment was performed with MAFFT with default parameters (3). Phylogenetic analysis was conducted with FastTree 2.1.11 (4) implemented in Geneious with default parameters using whole genomes available at GISAID (www.gisaid.org), including six other previously deposited Malaysian strains (EPI_ISL_416829, EPI_ISL_416866, EPI_ISL_416884, EPI_ISL_416885, EPI_ISL_416886, and EPI_ISL_416907).

**TABLE 1:** Comparison of nucleotide and amino acid differences among Malaysian strains

Malaysian strain	NGS^*a*^ (iSeq)						Mutation in gene at indicated position^*b*^															
	No. of raw reads	No. of mapped reads	Maximum coverage (×)	Avg coverage (×)	Genome size (bp)	GC content (%)	ORF1a					ORF1b			S	M	ORF8	N	3 UTR^*c*^			
Nucleotide position (in the genome)							2737	6310	6312	8782	11083	13730	13975	19524	23929	27147	28144	28311	29862	29868	29869	29871
Codon position (in the protein)							824	2015	2016	2839	3606	88		2019	789	209	84	13				
Reference SARS-CoV-2 (MN908947)					29,903	38.0	T (T)	C (S)	C (T)	C (S)	G (L)	C (A)	G (G)	C (L)	C (Y)	G (D)	T (L)	C (P)	G	T	G	C
hCoV-19/Malaysia/MKAK-CL-2020-5045/2020|EPI_ISL_416829							T (T)	C (S)	C (T)	C (S)	G (L)	C (A)	G (G)	C (S)	C (Y)	C (H)	T (L)	C (P)	G	T	G	C
hCoV-19/Malaysia/MKAK-CL-2020-5049/2020|EPI_ISL_416884							T (T)	C (S)	C (T)	C (S)	G (L)	C (A)	G (G)	C (S)	C (Y)	C (H)	T (L)	C (P)	G	T	G	C
hCoV-19/Malaysia/MKAK-CL-2020-5047/2020|EPI_ISL_416866							T (T)	C (S)	C (T)	C (S)	G (L)	C (A)	G (G)	C (S)	C (Y)	C (H)	T (L)	C (P)	G	T	G	C
hCoV-19/Malaysia/MKAK-CL-2020-6430/2020|EPI_ISL_416886							T (T)	C (S)	C (T)	T (S)	G (L)	C (A)	G (G)	C (S)	C (Y)	G (D)	C (S)	T (L)	G	T	G	C
hCoV-19/Malaysia/MKAK-CL-2020-5096/2020|EPI_ISL_416885							T (T)	C (S)	C (T)	T (S)	G (L)	C (A)	G (G)	C (S)	C (Y)	G (D)	C (S)	T (L)	G	T	G	C
hCoV-19/Malaysia/MKAK-CL-2020-7554/2020|EPI_ISL_416907							T (T)	C (S)	C (T)	C (S)	G (L)	C (A)	G (G)	C (S)	C (Y)	G (D)	T (L)	T (L)	G	T	G	C
*hCoV-19/Malaysia/186197/2020/EPI_ISL_417919*	1,467,222	369,427	73,335	1,087	29,486	36.6	**A (T)**	C (S)	C (T)	C (S)	G (L)	C (A)	G (G)	C (S)	C (Y)	G (D)	T (L)	T (L)	G	A	A	A
*hCoV-19/Malaysia/188407/2020|EPI_ISL_417918*	2,057,020	1,952,563	20,231	5,696	29,898	37.9	T (T)	**A (R)**	A (K)	C (S)	T (F)	T (V)	G (G)	**T (S)**	T (Y)	G (D)	T (L)	T (L)	G	A	A	A
*hCoV-19/Malaysia/190300/2020|EPI_ISL_417920*	1,796,760	1,647,233	30,677	4,986	29,865	37.6	T (T)	C (S)	A (K)	C (S)	T (F)	T (V)	G (G)	**T (S)**	T (Y)	G (D)	T (L)	T (L)	A	A	A	A
*hCoV-19/Malaysia/189332/2020|EPI_ISL_417917*	1,895,160	1,821,267	17,208	5,289	29,868	37.9	**A (T)**	**A(R)**	A (K)	C (S)	T (F)	T (V)	**A (S)**	**T (S)**	T (Y)	G (D)	T (L)	T (L)	A	A	A	A

a NGS, next-generation sequencing.

b Amino acids are denoted in parentheses; the four genomes of the second wave (sequenced in the present study) are italicized. Unique mutations found only in Malaysian strains (as of 29 March 2020) are bold.

c UTR, untranscribed region.

The four complete genome sequences reported here date from the main second wave of infections in Malaysia (Fig. 1A). Strain 188407 was linked to a religious mass gathering which has been associated with 48% of national cases and clusters with strains from Japan, Australia, and Saudi Arabia (Fig. 1B). Strain 189332 clusters with strain 188407, but the patient from whom it was isolated had no clear link to the gathering. This suggests that the strains associated with the gathering have established community transmission. The person with strain 186197 had travelled to Vietnam, while strain 190300, from a patient with no history of travelling or attending gatherings, was clustered with strains from Europe (Fig. 1B). Compared to reference strain Wuhan-Hu-1, Malaysian sequences have 16 nucleotide substitutions (Table 1). Four substitutions in the nonstructural region (ORF1a-T2737A, ORF1a-C6310A, ORF1b-G13975A, and ORF1b-C19524T) are unique to Malaysia, suggesting a degree of local evolution.

Our data showed that current circulating strains in Malaysia represent introductions from different countries and local evolution. More genomic data will clarify virus spread in Malaysia, particularly with respect to the role played by the mass gathering.

### Data availability.

These sequences have been deposited in the GISAID EpiCoV newly emerging coronavirus SARS-CoV-2 platform under identifiers EPI_ISL_417917 to EPI_ISL_417920. The sequences were also deposited in the following NCBI databases: GenBank (accession numbers MT372480 to MT372483), BioProject (PRJNA616147), BioSample (SAMN14483189, SAMN14483190, SAMN14596408, and SAMN14596409), and SRA (SRR11514750, SRR11514749, SRR11542244, and SRR11542243 [Illumina raw reads] and SRR11547279 [Nanopore raw reads]).
